# Effectiveness of intramuscular gluteal glucocorticoid injection versus intra-articular glucocorticoid injection in knee osteoarthritis: design of a multicenter randomized, 24 weeks comparative parallel-group trial

**DOI:** 10.1186/s12891-020-03255-9

**Published:** 2020-04-11

**Authors:** Marianne F. Mol, Jos Runhaar, P. Koen Bos, Desirée M. J. Dorleijn, Marijn Vis, Jacobijn Gussekloo, Patrick J. E. Bindels, Sita M. A. Bierma-Zeinstra

**Affiliations:** 1 Department of General Practice, Erasmus MC University Medical Center Rotterdam, PO-box 2040, 3000 CA Rotterdam, The Netherlands; 2 Department of Orthopaedic Surgery, Erasmus MC University Medical Center Rotterdam, Rotterdam, The Netherlands; 3 Department of Orthopaedic Surgery, University of Groningen, University Medical Center Groningen, Groningen, The Netherlands; 4 Department of Rheumatology, Erasmus MC University Medical Center Rotterdam, Rotterdam, The Netherlands; 5 Department of Public Health and Primary Care, Leiden University Medical Center, Leiden, The Netherlands; 6 Section of Gerontology and Geriatrics, Department of Internal Medicine, Leiden University Medical Center, Leiden, the Netherlands

**Keywords:** Knee osteoarthritis, Glucocorticoid injection, Intra-articular, Intramuscular, Randomized controlled trial, Non-inferiority, Primary care, General practice

## Abstract

**Background:**

The knee is symptomatically the most frequent affected joint in osteoarthritis and, in the Netherlands and other Western countries, is mainly managed by general practitioners (GPs). An intra-articular glucocorticoid injection is recommended in (inter) national guidelines for patients with knee osteoarthritis as an option for a flare of knee pain and/or for those who are not responding well to pain medication. An innovative approach that could replace the intra-articular injection is an intramuscular gluteal glucocorticoid injection. An intramuscular injection is easier to perform than an intra-articular injection with lesser risk of severe local adverse reactions. We hypothesize that intramuscular gluteal glucocorticoid injection is non-inferior in reducing knee pain compared to intra-articular glucocorticoid injection, with potentially a longer lasting effect than intra-articular injection.

**Methods/design:**

The study will be a pragmatic randomized controlled non-inferiority trial with two parallel groups. A total of 140 patients aged 45 years and older with knee osteoarthritis who contacted their general practitioner and have persistent knee pain (score ≥ 3 on 0–10 numerical rating scale; 0 = no knee pain) will be included.

Patients will be randomly allocated (1:1) to an injection of 40 mg triamcinolone acetonide intra-articular in the knee joint or intramuscular in the ipsilateral ventrogluteal area.

The effect of treatment will be evaluated by questionnaires at 2, 4, 8, 12, and 24 weeks after injection. The primary outcome is patients’ reported severity of knee pain measured with the pain subscale of the Knee injury and Osteoarthritis Outcome Score 4 weeks after injection. Statistical analysis will be based on both the per-protocol and the intention-to-treat principle.

**Discussion:**

This study will evaluate non-inferiority of intramuscular glucocorticoid injection compared to intra-articular glucocorticoid injection for knee osteoarthritis symptoms.

**Trial registration:**

This trial is registered in the Dutch Trial Registry (number NTR6968) at 2018-01-22 (https://www.trialregister.nl/trial/6784). Issue date: 1 October 2019.

**Trial sponsor:**

Erasmus MC University Medical Center Rotterdam.

PO-box 2040.

3000 CA Rotterdam.

The Netherlands.

## Background

The knee is symptomatically the most frequent affected joint in osteoarthritis (OA). In the Netherlands and other Western countries this is mainly managed by general practitioners [1]. The prevalence of knee OA in general practice was estimated around 40.2 per 1000 patient years (28.9 men; 51.4 women) in 2018 [2].

For OA patients, pain and disability are the most important reasons to seek care of a health professional [3–5]. If patients are not responding satisfactorily to paracetamol, NSAIDs and non-drug treatment, or in cases of interim aggravation, the evidence-based guideline from the Dutch College of General Practitioners on ‘Non-traumatic knee complaints’ suggests intra-articular (IA) glucocorticoid injection with 20 to 40 mg triamcinolone acetonide [6]. In several international guidelines IA glucocorticoid injection is also recommended for these abovementioned indications [7, 8].

Despite the long-standing frequent application of IA glucocorticoids, there is an ongoing debate about their effectiveness and safety [9, 10]. IA glucocorticoid injection in patients with knee OA leads to a moderate improvement in pain, but only in the short term (1 to 6 weeks after injection) [9]. IA injection has a small risk of the serious adverse reaction of septic arthritis on the short term [11]. In recent literature there is controversy over the chondrotoxicity of IA glucocorticoids on the longer term [12–14].

An additional obstacle in the way of IA injection is that GPs might feel incompetent to administer this type of injection due to lack of training and experience [15]. Due to the GP’s restraint, knee OA patients who could benefit from IA injection might not always receive timely injection [15].

Intramuscular (IM) glucocorticoid injection could be a valuable alternative treatment for IA glucocorticoid injection for patients with knee OA. IM administration eliminates the risk of septic arthritis and direct cartilage toxicity. The favorable effect of IM glucocorticoids on musculoskeletal pain has been studied originally in patients with rotator cuff disease and is used for rheumatoid arthritis [16, 17]. In a recent study from our study group, a clinical relevant and statistical significant difference in pain reduction was found for IM glucocorticoid injection compared to placebo in patients with hip OA [18]. Remarkably, the clinically relevant effect of the IM injection lasted at least 12 weeks. As of now no direct comparison between the effectiveness of IM and IA glucocorticoid injection in knee OA has been made.

We will perform a randomized controlled two-parallel-groups trial in patients with knee OA included from general practices, assessing the non-inferiority of an IM gluteal glucocorticoid injection compared to an IA glucocorticoid knee injection at 4 weeks follow-up. We hypothesize that IM gluteal glucocorticoid injection is non-inferior to IA glucocorticoid injection in reducing knee pain 4 weeks after injection, with potentially a longer lasting effect for at least 12 weeks.

### Primary objective

The primary objective is to assess whether IM gluteal glucocorticoid injection is non-inferior to IA knee glucocorticoid injection in reducing knee pain, measured with the Knee injury and Osteoarthritis Outcome Score (KOOS) pain subscale, in patients with knee OA in general practice at 4 weeks after injection.

### Secondary objectives

The study will evaluate the differences in reported adverse events frequency and co-interventions of patients allocated to an IM gluteal glucocorticoid injection or to an IA glucocorticoid injection. Differences between the two treatment groups in several outcome measures related to knee recovery, on short and longtime follow-up (2–24 weeks) and quality of life will be measured (see Table 2).

## Methods

### Design

**Fig. 1:**
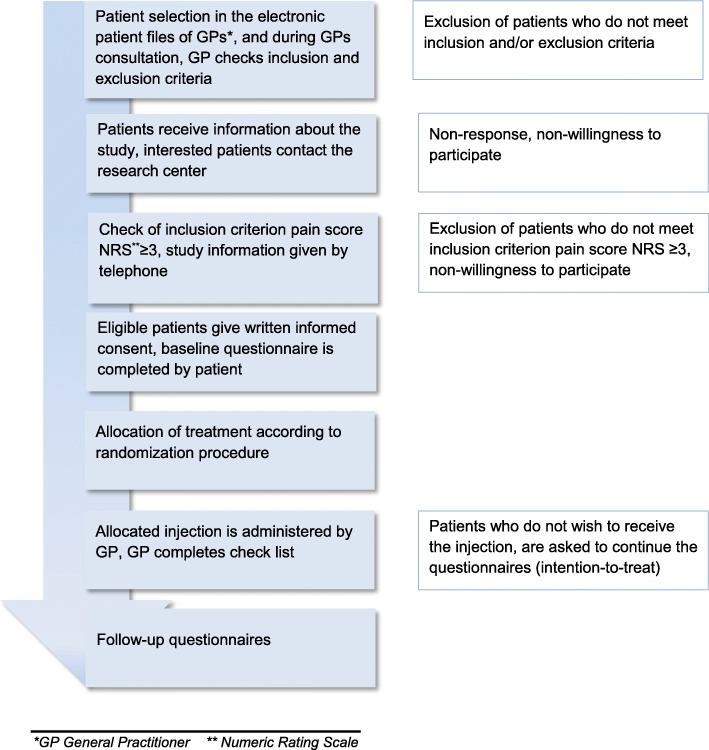
Flow-chart of patient selection

This study is a pragmatic randomized controlled non-inferiority trial with two parallel groups with a follow-up of 24 weeks (see Fig. 1). The Medical Ethics Committee of Erasmus MC University Medical Center Rotterdam approved this trial (MEC 2017–563). Any modifications to the protocol which may impact on the conduct of the study, potential benefit of the patient or may affect patient safety, including changes of study objectives, study design, patient population, sample sizes, study procedures, or significant administrative aspects will require a formal amendment to the protocol. Such amendment will be approved by the Medical Ethics Committee of Erasmus MC University Medical Center Rotterdam prior to implementation. All patients will give written informed consent prior to data collection.

### Patient selection

Patients with OA of the knee will be recruited by participating GPs located in the South-West of the Netherlands. Patient selection can take place in two different ways. The electronic patient files will be scanned for the International Classification of Primary Care (ICPC) codes L90 (knee OA) and L15 (knee symptoms/complaints) in order to identify eligible patients who have no contraindications for participation in this study. See Table 1 for the inclusion- and exclusion criteria. Patients who are identified with the ICPC code L15 are only selected if there is a note by the GP or a radiology report that mentions ‘osteoarthritis’ or ‘cartilage degeneration’.

**Table 1:** Eligibility criteria

Inclusion criteria: 1) contacted their GP^a^ (consultation and/or repeat pain medication prescription) due to knee OA^b^ (ICPC^c^ L90 or L15) during the past 5 years; 2) aged 45 years and over; 3) symptomatic knee OA for at least 3 months prior to enrolment; 4) a minimum score of 3 on the NRS^d^ asking about the severity of knee pain averaged over the past week (0–10; 0 = no knee pain); 5) glucocorticoid injection is indicated in this patient	
**Exclusion criteria:**	
1) use of oral glucocorticoids; 2) intra-articular injection in a knee in the previous 6 months; 3) allergy to glucocorticoids; 4) local or systemic infection, recent vaccination with live attenuated vaccine; 5) type 1 diabetes mellitus, type 2 diabetes mellitus on insulin therapy, poorly controlled type 2 diabetes mellitus; 6) presence of inflammatory rheumatic diseases (such as rheumatoid arthritis, psoriatic arthritis, spondylartropathies); 7) coagulopathy, use of anticoagulants, use of dual antiplatelet therapy; 8) a history of gastric/duodenal ulcer or a present gastric/duodenal ulcer;	
9) currently receiving care of an orthopaedic surgeon for OA of the hip and/or knee; 10) incapacity to complete questionnaires in Dutch; 11) incapacity to give informed consent.	

^a^GP General Practitioner ^b^OA Osteoarthritis ^c^ICPC International Classification of Primary Care ^d^Numeric Rating Scale

The second way is that GPs are asked to invite patients who consult them for knee OA to participate in the study. The GPs are asked to screen the inclusion- and exclusion criteria for all patients and will also directly assess whether there is an indication for glucocorticoid injection. An intra-articular glucocorticoid injection is recommended in guidelines for patients with knee osteoarthritis as an option for a flare of knee pain and/or for those who are not responding well to pain medication. Patients who have had an IA injection in the knee during the previous 6 months will be excluded, since a prolonged treatment effect of 24 weeks after injection has been described [19]. Patients with diabetes mellitus on insulin therapy or with a poor glycemic control (as assessed by their GP) cannot participate, as they might be at risk of prolonged blood glucose level elevation after glucocorticoid injection [20, 21]. Patients who have been referred to an orthopedic surgeon will also be excluded from participation considering that these patients could become candidates for total knee or hip arthroplasty during the follow-up period of the study. The risk of periprosthetic joint infection (PJI) is increased in patients who received an IA glucocorticoid injection in the 3 months prior to arthroplasty [22, 23]. It is not known if IM glucocorticoid injection increases the risk of PJI.

For patients who are selected via their electronic patient file, the actual amount of knee pain they experience is not known by their GP. These patients’ knee pain level will be checked over the phone by the researchers (given that the patient is willing to participate in the study, see Fig. 1). A minimum score of 3 on the numerical rating scale (NRS, 0–10; 0 = no knee pain) is required in order to participate in the trial. If a patient has an NRS knee pain score < 3, the patient will be asked to contact the research team in case of future increase in knee pain. In case a patient has bilateral knee OA, the most painful knee is selected as the ‘study knee’.

### Procedures

The GPs will inform all eligible patients about the study in writing. Patients will receive information about the study and a reply card. Once the research team receives a reply card with a positive response from a patient, a researcher will contact this patient by telephone. The researcher will ask the patient about the severity of knee pain averaged over the past week. To all patients with a pain score ≥ 3 additional written information about the study will be sent. Some days later, the researcher or trained research assistant will contact the patients again to further explain the study and to answer remaining questions. Patients who are interested to participate will be asked to give written informed consent. After the patients have given this consent, the baseline questionnaire is sent to these patients.

After completion of the baseline questionnaire, the patient will be randomly allocated to one of the two treatment groups. The GP and the patient will be informed about the outcome of randomization. The GP will prepare and administer the allocated injection. We aim to have the injection administered within 1 week after completion of the baseline questionnaire. This is to ascertain the pain score at the moment of injection is unchanged or close to the baseline score. Change in pain since baseline can never lead to exclusion of a trial participant.

The GP will complete a case report form at the patients’ visit for administration of the injection. This report form asks for the American College of Rheumatology criteria for clinical diagnosis of knee OA, location of injection, severity of knee pain averaged over the past week and the batch number of the triamcinolone acetonide [24].

All patients will be referred for an AP weight-bearing X-ray of the studied knee if an X-ray has not been made in the 12 months prior to enrolment. The 12 month period was chosen as the risk of annual radiographic OA progression by at least one Kellgren-Lawrence (K-L) grade has been estimated low [25]. Therefore, the X-ray does not have to be obtained directly at baseline, but will be made during follow-up. We consider the radiograph necessary in order to facilitate comparison between our study population and patient data collected in previous studies. Two researchers will independently assess the X-rays to grade radiographic knee OA, using the K-L classification [26].

### Randomization

After the patients sign informed consent, they will be randomized and receive their allocated intervention. After informed consent the patient will be assigned a unique trial number.

An independent researcher, who will not meet or contact the patients, has prepared a computer generated randomization list using 1:1 allocation and random blocks of 8, 6 or 4 in order to ensure concealment of allocation.

### Blinding

Due to the pragmatic nature of this trial, the patient and the GP are not blinded for treatment allocation. The researcher involved in data analysis will be blinded for treatment allocation.

### Intervention

The investigational treatment will consist of 40 mg triamcinolone acetonide (Kenacort-A 40). The chosen dosage of glucocorticoid is based on clinical experience [27]. No local anesthetic will be added to the injection. The GP will inject either IA in the knee joint or IM in the ipsilateral ventrogluteal region. All participating GPs will be invited for an optional IA knee injection training under supervision of an experienced orthopedic surgeon (PKB).

**Fig. 2:**
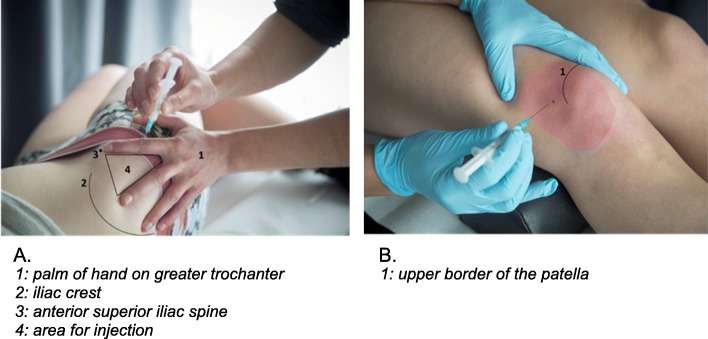
Ventrogluteal intramuscular and superolateral intra-articular injection techniques

The superolateral IA injection approach to the knee will be used (just below the upper border of the patella and 1 cm lateral to the lateral border of the patella, see Fig. 2b). This approach has an accuracy of 91% for needle placement in the IA space of the knee and is recommended by the Dutch College of GPs [6, 28].

The IM injection will be administered in the ventrogluteal region (the region between the iliac crest, greater trochanter of the femur and anterior iliac spine, see Fig. 2a) in order to prevent injury to the sciatic nerve [29, 30]. Moreover, administration in the ventrogluteal region diminishes the possibility of subcutaneous injection in overweight patients since the layer of subcutaneous fat is less thick in the ventrogluteal region compared to the dorsogluteal region [31]. It is well known that knee OA is more common in patients with a BMI > 27 kg/m^2^ [32].

Co-interventions are allowed during the follow-up and will be monitored.

### Measurements

#### Baseline measurements

See Table 2 for an overview of baseline measurements. Demographic measurements consist of age, sex, educational level, and daily occupation. Also self-reported length and weight are reported. Medication use for knee OA will be measured using a multiple choice format where patients can select multiple answers and add a missing medicament.

**Table 2:** Scheduled measurements of primary and secondary outcomes

Measurement	Baseline	Injection	2 weeks fu	4 weeks fu	8 weeks fu	12 weeks fu	24 weeks fu
***Primary outcome measure***							
KOOS pain subscale	x		x	x	x	x	x
***Secondary outcome measures***							
Adverse events			x				
Hospitalization			x	x	x	x	x
Co-interventions (iMCQ)			x	x	x	x	x
Medication use for knee OA	x		x	x	x	x	x
Re-injection with glucocorticoid					x	x	x
KOOS stiffness	x		x	x	x	x	x
KOOS function in daily living	x		x	x	x	x	x
KOOS sports and recreation	x		x	x	x	x	x
KOOS QoL	x		x	x	x	x	x
ICOAP	x		x	x	x	x	x
OMERACT OARSI responder criteria			x	x	x	x	x
Knee pain over past week (NRS)	x	x	x	x	x	x	x
Perceived recovery (Likert scale)			x	x	x	x	x
Knee complaint characteristics	x		x	x	x	x	x
Health related QoL	x						
***Additional measurements***							
Radiograph of study knee						x	
Check of ACR criteria		x					
Painfulness of injection (NRS)			x				
Demographic information	x						
Co-morbidity	x						
Physical activity over the past week (IPAQ short)	x						
Neuropathic pain (Modified painDETECT Questionnaire)	x						
Patients’ preferred injection site	x						
Patients’ expected treatment response	x						

Intermittent and constant OA pain will be measured with the Intermittent and Constant OsteoArthritis Pain score (ICOAP:0–100; 0 = no pain) [33]. Knee complaint characteristics (duration of symptoms at baseline, sensation of swelling in the knee as an indicator of flare-up) will be recorded. Knee pain severity averaged over the last week will be measured with an 11-point numerical rating scale (NRS:0–10; 0 = no pain). Health related Quality of Life (QoL) will be measured with the EQ-5D-5 L (scores ranging from − 0.446 = worst health related QoL to 1.0 = perfect health related QoL) [34, 35] Co-morbidity will be measured at baseline using a multiple choice format where patients can select multiple answers and add a missing comorbid disease. Also measured at baseline will be physical activity over the past week (IPAQ short), neuropathic pain (modified painDETECT questionnaire), patients’ preferred injection site (knee or ventrogluteal area) and patients’ expected treatment response [36, 37].

#### Follow-up measurements

Outcomes are measured at 2, 4, 8, 12 and 24 weeks after administration of the injection using digital questionnaires. Patients without an electronic mailbox will receive paper questionnaires. The primary outcome is patient reported severity of pain at 4 weeks after injection measured with the KOOS pain subscale (0–100; 0 = extreme pain). Secondary study endpoints are listed in Table 2. Patients’ perceived recovery is measured with a 7-point Likert scale that will be dichotomized in recovered (‘complete recovery’, ‘much improved’, ‘slightly improved’) and not-recovered (‘no change’, ‘slightly worse’, ‘much worse’,“worse than ever’) [18]. Percentage responders is defined by the OMERACT-OARSI criteria: High improvement (≥50%) in KOOS pain subscale or in KOOS function in daily living subscale and absolute increase ≥20 points in KOOS pain subscale or function in daily living subscale, if not then improvement in at least 2 of the 3 following domains: 1) ≥20% improvement in KOOS pain subscale and ≥ 10 points increase in KOOS pain subscale, 2) ≥20% improvement in KOOS function in daily living subscale and ≥ 10 points increase in KOOS function in daily living subscale, 3) ≥20% increase in global score and ≥ 10 points increase in global score. In this study patients’ global score will be measured with a patients’ perceived recovery score measured on a 7-point Likert scale. This domain is considered improved if a patient fills in ‘complete recovery’, ‘much improved’, or ‘slightly improved’ [38]. Two weeks after administration of the injection patients are asked to report adverse events. Also, follow-up questionnaires at all time points ask about hospitalization to monitor Serious Adverse Events. Co-interventions including medication, non-drug therapies such as physiotherapy, referrals and surgery will be measured with the modified medical consumption questionnaire of the Institute for Medical Technology Assessment (iMCQ) [39]. Experienced painfulness of injection will be registered 2 weeks after glucocorticoid injection. All data will be handled according to the Data Monitoring Plan, as drafted and approved by the funder in preparation of data collection.

### Sample size

For the sample size calculation, we used data from the study of Henriksen et al. that evaluated the clinical benefits of an IA glucocorticoid injection given before exercise therapy in patients with knee OA [40]. The results of the study reported a baseline standard deviation of 16 for the KOOS pain (recommended by the KOOS for sample size calculations is a standard deviation of 15). The minimal important difference (=non-inferiority margin) between both treatment groups of the patient reported outcome KOOS (0–100) was set at 7 points (effect size of 0.44) [41, 42].

For the non-inferiority of an IM gluteal glucocorticoid injection compared to an IA knee glucocorticoid injection, we will need 65 patients per group, using a power of 80%, an alpha of 5%, a non-inferior margin of 7 and a SD of 16. Taking into account a loss to follow-up of 5%, this trial needs to include (2*65) + (0.05*2*65) = ~ 140 patients. We expect a low percentage of loss to follow-up because of the relative short follow-up period of 24 weeks and our prior experience in glucocorticoid trials [18, 43].

### Data analyses

Imbalance in the baseline variables of the two treatment arms might occur after randomization. This is problematic if the imbalanced variable is related to the outcome variable, as this could lead to confounding [44, 45]. In case imbalance occurs, we will adjust for relevant variables. Descriptive statistics will be used to describe patients’ and complaints characteristics at baseline. Analyses will be adjusted for variables that hamper the baseline interchangeability of groups when there are clinically relevant differences between groups of over 10%.

The primary outcome is patient reported severity of pain at 4 weeks after injection measured with the KOOS pain (0–100; 0 = extreme pain). We will use a non-inferiority design to assess if an IM gluteal injection is non-inferior to an IA knee injection with regard to this outcome. In non-inferiority comparisons intention-to-treat analysis can bias towards the null and could increase type I error; the risk of falsely claiming non-inferiority [46]. Therefore, the per-protocol analysis will be the primary analysis. Included in the per-protocol analysis will be patients who received the assigned injection and reported the KOOS pain at 4 weeks follow-up. In case a patient from the IM injection group receives an additional IA injection earlier than 6 weeks after the study injection, this will be considered as a protocol violation and the patient will be excluded from the per-protocol analysis. The reason for this is that the guideline from the Dutch College of General Practitioners recommends to leave at least 6 weeks between two consecutive injections [6]. Non-inferiority of the IM injection will also be assessed using both intention-to-treat and per-protocol analysis at 2, 8, 12 and 24 weeks follow-up and for the outcome KOOS pain. For the other outcome measures we will calculate mean differences.

We expect 10–15% of missing data (incompletely filled in paper questionnaires and loss to follow-up). We will contact the patients to pose them the missing questions again in order to minimize missing data for the primary outcome. Multiple imputations will be performed for missing values (incompletely filled in questionnaires), creating at least five imputed datasets.

Linear mixed models with repeated measures will be used to calculate group differences over time for the primary outcome as this is a continuous variable. To model the covariance of repeated measures by patients, a structure will be chosen with the lowest Akaike’s information criterion. Fixed effects will be time, and time by treatment.

Linear mixed models with repeated measures will also be used for the continuous secondary outcomes: KOOS, NRS, WOMAC, ICOAP, and EQ-5D-5 L. Generalized estimating equations analyses with repeated measures will be performed for the dichotomous outcomes: patients’ perceived recovery (7 point Likert scale), and the OMERACT-OARSI responder criteria. Before generalized estimating equations analyses, multiple imputations will be performed for missing values of secondary study parameters, creating at least five imputed datasets.

When patients underwent a total knee replacement surgery, data of these patients will be included up to the date of surgery. Missing data for secondary outcome measurements will be handled similarly as missing data for the primary outcome.

### Subgroup analysis

An explorative, pre-defined, subgroup analysis will be performed assessing the interaction effects between injections regarding the severity of knee pain at baseline (NRS pain score of > = 7 versus < 7) on the primary outcome [47]. Generalized estimating equations will be used to analyze differences between groups concerning adverse events, medical consumption and medication usage.

## Discussion

This study will evaluate non-inferiority of intramuscular glucocorticoid injection compared to intra-articular glucocorticoid injection for knee osteoarthritis symptoms, during 24 weeks of follow-up. The primary outcome is the patient reported severity of pain at 4 weeks after injection, using a non-inferiority margin of 7 points on the patient reported outcome KOOS between both treatment groups.

The sample size calculations indicated a required sample of 140 participants, taking into account a loss to follow-up of 5%. Recruitment was finalized on February 11, 2020.

### Strength and limitations

The current study is a high-quality pragmatic trial, which will provide a reliable comparison between intra-articular and intramuscular corticosteroid injections for individuals with knee OA and will make implementation of its results highly feasible. Obviously, patients will not be blinded to the location of the injection in the current study. Although this might bias the results towards the by the patients preferred injection location (patients’ preference will be assessed), we already know from our previous study on hip OA [18] that the intramuscular corticosteroid injection was superior to placebo. So the clinical effectiveness of the intramuscular corticosteroid injection did not rely on contextual effects only.

## Data Availability

Not applicable.

## Abbreviations

GP: General Practitioner
IA: intra-articular
ICOAP: Intermittent and Constant OsteoArthritis Pain
ICPC codes: International Classification of Primary Care (ICPC) codes
IM: Intramuscular
iMCQ: Institute for Medical Technology Assessment
K-L grade: Kellgren-Lawrence grade
KOOS: Knee injury and Osteoarthritis Outcome Score
NRS: numerical rating scale
OA: osteoarthritis
PJI: periprosthetic joint infection
QoL: Quality of Life
